# Quantitative profiling of m^6^A at single base resolution across the life cycle of rice and *Arabidopsis*

**DOI:** 10.1038/s41467-024-48941-7

**Published:** 2024-06-07

**Authors:** Guanqun Wang, Haoxuan Li, Chang Ye, Kayla He, Shun Liu, Bochen Jiang, Ruiqi Ge, Boyang Gao, Jiangbo Wei, Yutao Zhao, Aixuan Li, Di Zhang, Jianhua Zhang, Chuan He

**Affiliations:** 1 Department of Chemistry, The University of Chicago, Chicago, IL 60637 USA; 2 Department of Biochemistry and Molecular Biology, The University of Chicago, Chicago, IL 60637 USA; 3 Institute for Biophysical Dynamics, The University of Chicago, Chicago, IL 60637 USA; 4 Howard Hughes Medical Institute, Chicago, IL 60637 USA; 5 Department of Biology, Hong Kong Baptist University and School of Life Sciences and State Key Laboratory of Agrobiotechnology, The Chinese University of Hong Kong, Shatin, Hong Kong; 6 Present Address: Department of Chemistry and Department of Biological Sciences, National University of Singapore, Singapore, Singapore

**Keywords:** RNA, RNA modification, Plant development

## Abstract

*N*^6^-methyladenosine (m^6^A) plays critical roles in regulating mRNA metabolism. However, comprehensive m^6^A methylomes in different plant tissues with single-base precision have yet to be reported. Here, we present transcriptome-wide m^6^A maps at single-base resolution in different tissues of rice and *Arabidopsis* using m^6^A-SAC-seq. Our analysis uncovers a total of 205,691 m^6^A sites distributed across 22,574 genes in rice, and 188,282 m^6^A sites across 19,984 genes in *Arabidopsis*. The evolutionarily conserved m^6^A sites in rice and *Arabidopsis* ortholog gene pairs are involved in controlling tissue development, photosynthesis and stress response. We observe an overall mRNA stabilization effect by 3’ UTR m^6^A sites in certain plant tissues. Like in mammals, a positive correlation between the m^6^A level and the length of internal exons is also observed in plant mRNA, except for the last exon. Our data suggest an active m^6^A deposition process occurring near the stop codon in plant mRNA. In addition, the MTA-installed plant mRNA m^6^A sites correlate with both translation promotion and translation suppression, depicting a more complicated regulatory picture. Our results therefore provide in-depth resources for relating single-base resolution m^6^A sites with functions in plants and uncover a suppression-activation model controlling m^6^A biogenesis across species.

## Abstract (web summary)

The authors profile single-base resolution maps at each modified mRNA m^6^A site in rice and Arabidopsis, and uncover a suppression-activation dual regulation model in shaping m^6^A distribution patterns in different species.

## Introduction

RNA modifications are critical regulators of mRNA processing and metabolism, which include splicing, 3′-end processing, nuclear export, translation, and decay. m^6^A is identified as the most abundant internal mRNA modification in mammals and plants^1–12^. In *Arabidopsis*, two different m^6^A writer complexes have been suggested to install m^6^A on mRNA. The first methyltransferase complex is composed of five respective orthologs of the components of the mammalian m^6^A methyltransferase complex that include mRNA adenosine methylase (MTA), MTB, VIRILIZER (VIR), FKBP12 INTERACTING PROTEIN 37KD (FIP37), and an E3 ubiquitin ligase HAKAI^13–16^. FIONA1 (FIO1), by contrast, is the *Arabidopsis* ortholog of the human methyltransferase METTL16, also depositing m^6^A modifications in U6 small nuclear RNA and a subset of mRNAs^17–19^. Defects in the m^6^A writer protein complex, such as MTA failure in plants, cause an embryo-lethal phenotype^15,20–22^ and stress responses^23–27^. Studies of other components of the writer complex in *Arabidopsis* revealed that FIP37 regulates shoot stem cell fate, FIO1 regulates floral transition and chlorophyll homeostasis, and VIRILIZER (VIR) is critical to vascular development^13,14,18,19,28^; whereas the defect of FIP37 in rice leads to early degeneration of microspores^29,30^. The m^6^A methylation can be reversed^31,32^. RNA m^6^A demethylases ALKBH10B and ALKBH9B, homologs of the human m^6^A demethylase ALKBH5^32^, affect floral transition^33^ and viral infection^34^ in *Arabidopsis*. In our recent study, overexpression of the mammalian m^6^A demethylase FTO in rice dramatically increased the biomass and yield of rice and potato^35^, revealing that modulation of RNA m^6^A methylation could be a promising breeding or engineering strategy for crop improvement in the future. Although these observations indicate conserved mRNA m^6^A methylation regulators in plants as compared with animals, so far the high-resolution mRNA m^6^A maps in plants are still missing and the molecular level connection of m^6^A to plant development and other pathways are mostly unknown. We proceeded to fill this gap by using the newly developed high-resolution sequencing method^36,37^.

Methylated RNA m^6^A immunoprecipitation sequencing (MeRIP-seq or m^6^A-seq)^38^ has been widely used in identifying m^6^A-enriched transcripts in animals and plants. However, this method lacks single-base resolution and cannot quantify the extent of the modification. Variations of MeRIP-seq, including m^6^A individual-nucleotide-resolution crosslinking and immunoprecipitation (miCLIP)^39^, have been developed to detect m^6^A sites at single-base resolution. Unfortunately, these methods typically display low efficiency of UV crosslinking and cannot assess modification stoichiometry. Antibody-independent single-base m^6^A profiling methods, such as m^6^A-REF-seq^40^ or MAZTER-seq^41^, have been introduced. However, these methods can only identify RNA modifications in the ACA motif, and fail to identify other methylation motif. Nanopore direct RNA-seq (DRS) has been utilized to map m^6^A sites, but quantifying the differences in m^6^A modification levels with DRS is still challenging^42–44^. We have recently reported m^6^A-selective allyl chemical labeling and sequencing (m^6^A-SAC-seq)^36,37^ as a method capable of precisely mapping of individual m^6^A-modified sites in whole transcriptomes at single-nucleotide resolution. Although there are recently reported deamination-based methods that can also map m^6^A at base resolution, including one from us and collaborators^45,46^, m^6^A-SAC-seq is capable of reading m^6^A as a positive mutation signal without subtraction, and the optimized protocol works with 2–5 ng of input RNA^36^. We, therefore, decided to deploy m^6^A-SAC-seq to establish comprehensive maps of mRNA m^6^A at single-nucleotide resolution across various tissue types in two different plant species: *Arabidopsis* and rice.

A considerable number of high-confident m^6^A sites were identified spanning the entire life cycle of both rice and *Arabidopsis*. The evolutionarily conserved m^6^A mRNA modification sites across rice and *Arabidopsis* ortholog gene pairs play regulatory roles in tissue development, photosynthesis, and stress response. m^6^A levels are positively correlated with the length of the internal exon, but such correlation is missing in the last exon. Through comparative base-resolution m^6^A analysis across humans, rice and *Arabidopsis*, we unveil a distinct m^6^A distribution pattern that a suppression-activation dual model governs the m^6^A deposition in humans and plants. Using the robust method for comparison of m^6^A levels at single base resolution, we noticed that rice and *Arabidopsis* possess higher percentages of the overall m^6^A modifications in the 3’ UTR of their mRNAs than those of mammalian systems. These 3’ UTR m^6^A modifications generally stabilize mRNA and enhance translation, and these effects correlate well with the m^6^A fraction. These observations indicate that both fraction and position of m^6^A modification are critical for mRNA metabolism in plants. In addition, we noticed that the MTA-mediates m^6^A deposition in photosynthesis-related genes that transcribed from both nuclear and chloroplast genome in *Arabidopsis*. These m^6^A sites can either promote or reduce translation efficiency in a pathway-dependent manner. Altogether, our base-resolution and quantitative m^6^A sites across rice and *Arabidopsis*, provide a foundation for future studies to explore the regulatory roles of m^6^A in regulating plant development and evolution and for future plant engineering.

## Results

### m^6^A-SAC-seq identifies m^6^A modification sites in rice and *Arabidopsis*

**Fig. 1:**
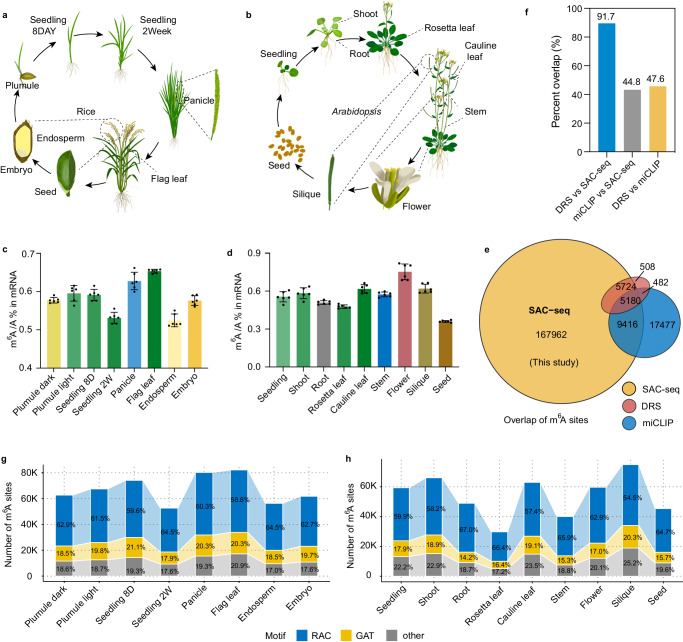
m^6^A-SAC-seq effectively identifies m^6^A sites across plant tissues. **a,** **b**, Tissues from eight different rice organs (**a**) and nine different *Arabidopsis* organs (**b**), collected throughout their respective life cycles, were subjected to m^6^A-SAC-seq. **c, d**, mRNA m^6^A levels in the harvested samples were quantified using LC-MS/MS for both rice (**c**) and *Arabidopsis* (**d**). The m^6^A-to-A ratio was determined using calibration standards. Data are means ± SD, *n* = 6. **e**, Venn diagram showing m^6^A sites detected by SAC-seq overlapping with those identified by miCLIP and DRS, with the ±5 nt sliding window around each m^6^A site. **f**, A comparison of the percentages of overlapping m^6^A sites identified by different methods. **g,** **h**, The number of m^6^A sites and their motif distribution in rice (**g**) and *Arabidopsis* (**h**) tissues were shown. Motif sequences were separated into three groups, RAC, GAT and others. Base “R” denotes either A or G. 8D represents 8 days and 2 W represents 2 weeks. Source data are provided as a Source Data file.

m^6^A-SAC-seq utilizes the dimethyltransferase MjDim1 to introduce an allyl group to m^6^A, which upon chemical-induced cyclization could be read as mutation signals during reverse transcription (Supplementary Fig. 1a)^36,37,47^. We extracted total RNAs from nine *Arabidopsis* tissues (seedling, shoot, root, rosette leaf, cauline leaf, stem, flower, silique and seed) as well as eight rice tissues (plumule dark, plumule light, seedling at 8 days, seedling at 2 weeks, panicle, flag leaf at 10 days after anthesis, endosperm at 10 days after anthesis, and embryo at 10 days after anthesis) with two biological replicates for each sample (Fig. 1a, b). PolyA-tailed RNA of each biological replicate was purified and subjected to LC-MS/MS to measure the m^6^A/A ratio. The ratio of m^6^A/A in polyA-tailed RNA from these different tissues varied within the range of 0.36–0.75% in *Arabidopsis* (Fig. 1c) and in the range of 0.52–0.67% in rice (Fig. 1d). The remaining polyA-tailed RNAs were then processed following the m^6^A-SAC-seq library construction protocol^36,37^ to map m^6^A sites at the base resolution.

To reduce background noise and eliminate any potential batch effect, we added 2% spike-in calibration probes into each sample. These calibration probes contained varying fractions of m^6^A within the NNm^6^ANN motif. By determining the mutation rates of both A and m^6^A within different motifs in these probes, we could best determine the m^6^A modification fraction at individual sites in each sample. Our initial results showed that the labeling efficiency of the calibration probes in plant cells was very high, comparable to that observed in mammalian cells (Supplementary Fig. 1b**)**. Additionally, the average background noise of unmodified A sites was as low as 0.49%, which is 10 times lower than the cutoff (<5%) used for m^6^A site detection, indicating high m^6^A-SAC-seq data quality obtained from these plant samples. Furthermore, we assessed the relative conversion ratio and background noise in each sample. The relative conversion ratio ranged from 0.92 to 1.11 in *Arabidopsis* samples (Supplementary Fig. 1c) and from 0.96 to 1.07 in rice samples (Supplementary Fig. 1d), showing consistent efficiency of m^6^A labeling across samples. By carefully calibrating the differences observed in each sample, we ensured a fair and accurate comparison of m^6^A levels among different samples. This calibration step is crucial for obtaining reliable and meaningful results, enabling us to effectively compare the m^6^A modification among various plant samples.

Before proceeding to downstream analysis, we compared our m^6^A-SAC-seq data with previously published m^6^A sites profiled by DRS^48^ and miCLIP^39^ in *Arabidopsis*. Only sites with a sufficient number of sequence coverage among all tissues (depth> 10) were selected for further analysis. A total of 188,282 m^6^A sites were obtained when we combined the m^6^A sites detected by SAC-seq in this study, with ~42% and ~17% of the m^6^A sites measured by DRS and miCLIP overlapping with SAC-seq m^6^A sites, respectively (Supplementary Fig. 1e). With a sliding window of ±5 nt around the m^6^A sites, many more previously detected m^6^A sites overlap with the m^6^A-SAC-seq sites (Fig. 1e, f). As expected, a much higher percentage of m^6^A sites overlap between m^6^A-SAC-seq and DRS than that between m^6^A-SAC-seq and miCLIP (Fig. 1f). This might suggest the low accuracy of miCLIP in identifying m^6^A site, which could be caused by low crosslinking efficiency in plant tissues.

Principal component analysis (PCA) revealed a distinct clustering based on the m^6^A fractions of the different tissues (Supplementary Fig. 2a–d). An average of 49,791 m^6^A sites from 12,652 genes with at least 20 reads were identified in *Arabidopsis* libraries. In rice, an average of 67,173 m^6^A sites were detected from 15,138 genes. The number of high-confidence m^6^A sites from eight different rice tissues varied within the range of 52,646 in seedling 2 W to 82,157 in flag leaf (Fig. 1g). While the number of m^6^A sites among nine *Arabidopsis* tissues ranged from 25,990 in seed to 74,259 in silique (Fig. 1h). In comparison with previously published *Arabidopsis* MeRIP data of seedling (7,489 m^6^A peaks), we identified approximately 59,212 m^6^A sites within 14,180 genes in the seedling datasets, demonstrating high sensitivity of the SAC-seq method. Therefore, *Arabidopsis* seedling transcriptome contains ~4.2 m^6^A sites per gene, which is four-fold higher than that observed in the MeRIP data^49^. Interestingly, ~4.4 m^6^A sites per gene were observed among rice tissues, suggesting a likely conserved distribution density across different plant species. Consistent with the findings in mammals^36,37^ and plants^9,50,38,51^, the RAC (R = A or G) motif displayed the highest frequency among methylated motifs in both rice and *Arabidopsis* (Fig. 1g, h).

### Base-resolution mRNA m^6^A maps from different tissues of rice and *Arabidopsis*

**Fig. 2:**
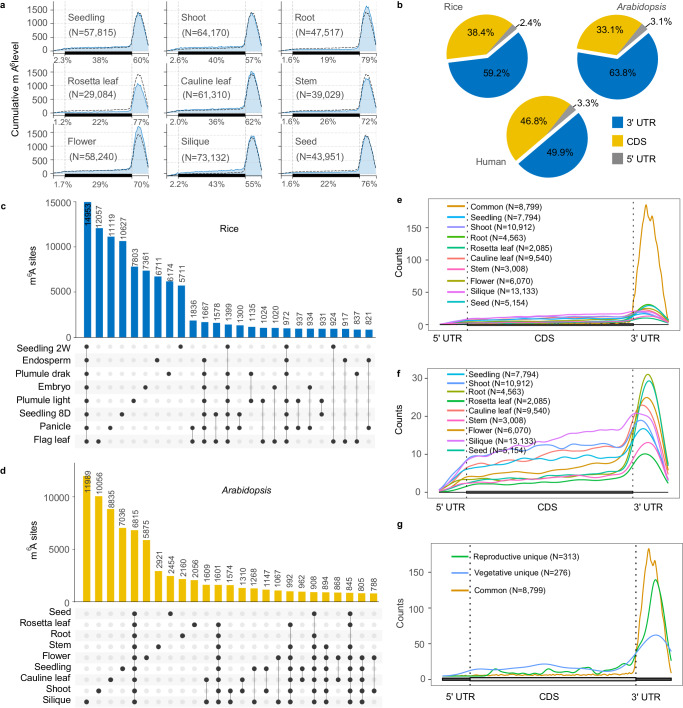
Comprehensive base-resolution maps of m^6^A sites in rice and *Arabidopsis.* **a** Metagene profiles showing the m^6^A site distribution across transcripts in nine *Arabidopsis* tissues. Each transcript is segmented into three regions: 5′ UTR, CDS and 3′ UTR. The black dashed line represents the average m^6^A fraction across the nine tissues. The m^6^A site number (N) is indicated in the figure. The percentage (%) of overall m^6^A modifications distributed in 5′ UTR, CDS, and 3′ UTR regions within different tissues were shown. **b** Percentages of total m^6^A modifications distributed in 5′ UTR, CDS, and 3′ UTR regions in Hela cells, rice and *Arabidopsis*. We combined m^6^A sites from all the rice tissues and *Arabidopsis* tissues, respectively, to calculate percentages of total m^6^A fractions within 5′ UTR, CDS, and 3′ UTR regions. **c,** **d** Bar plot showing the number of tissue-unique and tissue-common m^6^A sites in eight rice tissues (**c**) and nine *Arabidopsis* tissues (**d**). The m^6^A site number is the overlapped m^6^A sites between two biological replicates for each tissue. **e** Metagene profiles showing common m^6^A sites among nine *Arabidopsis* tissues distributed across transcripts. **f** Metagene profiles of tissue-unique m^6^A sites among nine *Arabidopsis* tissues. **g** Metagene profiles showing the rice reproductive unique-, vegetative unique- and common m^6^A sites distributed across transcripts. Tissues of flower, seed and silique were grouped as reproductive tissues, while the remaining tissues were considered vegetative. Each transcript is divided into three regions: 5′ UTR, CDS and 3′ UTR. For **e**.**g.**, the numbers of m^6^A sites are indicated in the figures.

We next analyzed the distribution of m^6^A in the whole transcriptome for both *Arabidopsis* and rice. All the identified m^6^A sites in *Arabidopsis* and rice are accessible under the GEO numbers GSE245738 and GSE243722, respectively. As observed in the metagene profile, most m^6^A sites are highly enriched within 3′-untranslated region (3′ UTR), followed by coding DNA sequence (CDS) and 5′-untranslated region (5′ UTR) in both *Arabidopsis* (Fig. 2a) and rice (Supplementary Fig. 3a**)**. Although both mammalian and plant mRNAs highly enrich m^6^A in the 3’ UTR (Fig. 2b**)**, rice and *Arabidopsis* mRNAs harbor noticeably higher percentages of the overall m^6^A modifications in the 3’ UTR compared with that of human HeLa cells^36^ (Fig. 2b**)**. In addition to m^6^A sites in the regions of 3’ UTRs and CDSs, we also observed a considerable number of m^6^A sites in the intronic, and 5’ UTRs regions in both rice and *Arabidopsis*, which are consist with past results observed in mammals^37^ (Supplementary Fig. 3b, c). The average m^6^A fraction is notably higher in intronic regions than in the 5’ UTR and CDS, but lower than that in the 3’ UTR (Supplementary Fig. 3b, c).

Both the m^6^A/A ratio and m^6^A site number vary among different tissues, suggesting the presence of both tissue-shared and tissue-specific deposition of m^6^A methylation in plants. We, therefore, analyzed tissue-specific and commonly shared m^6^A modification sites among all the tissues in rice and *Arabidopsis*, and identified 14,953 and 6,815 shared m^6^A sites among all rice and *Arabidopsis* tissues, respectively (Fig. 2c, d). These shared m^6^A modification sites were predominately enriched in the 3′ UTR region relative to this tissue-specific m^6^A both in rice (Supplementary Fig. 3d, e) and *Arabidopsis* (Fig. 2e, f), which may suggest that 3′ UTR m^6^A sites, rather than the CDS or 5′ UTR, play general roles in maintaining plant transcriptome metabolism across the entire life cycle. To find the biological difference between reproductive- and vegetative unique m^6^A sites, we further divided the different tissues into reproductive tissue and vegetative tissue (see Method). The reproductive unique and vegetative unique m^6^A sites were identified in both rice and *Arabidopsis*, respectively (Supplementary Data 1). The metagene profile revealed that reproductive unique m^6^A sites show increased distribution in the 3′ UTR region compared to the vegetative unique one, implying the significance of m^6^A regulation through 3′ UTR in the reproductive phase (Fig. 2g and Supplementary Fig. 3f). GO enrichment analysis showed that genes containing reproductive unique m^6^A sites in both rice and *Arabidopsis* are enriched in similar pathways, such as reproductive structure development, embryo development, immune response, photosynthesis, and chloroplast organization (Supplementary Fig. 3g). Meanwhile genes containing vegetative unique m^6^A modifications are enriched in stimulus response, such as genes of *ARF1*, *ARF7* and *ARF9* involved in response to hormone stimulus in rice (Supplementary Fig. 3h), although these pathways are not significant enriched in *Arabidopsis*. This likely suggests tissue-specific m^6^A methylations play regulatory roles in plant growth regulation.

### Evolutionary conservation and variability of m^6^A regulation across rice and *Arabidopsis*

**Fig. 3:**
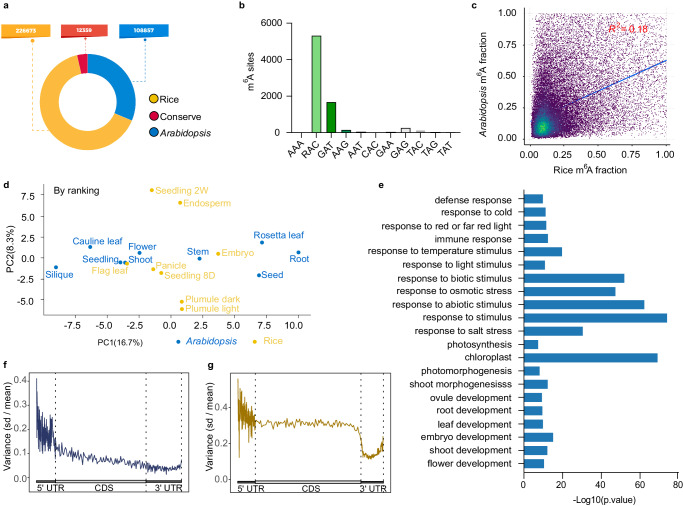
Evolutionary conservation and divergence in m^6^A regulation between rice and *Arabidopsis.* **a** Number of conserved and species-unique m^6^A sites between rice and *Arabidopsis*. **b** Motif of RAC is the most prevalent one among the conserved m^6^A sites. Motif sequences were divided into three groups, including RAC, GAT and others. Base “R” denotes either A or G. **c** Correlation analysis of the m^6^A fraction in the ortholog gene pair between rice and *Arabidopsis*. The *R*^*2*^ value is labeled in the figure. **d** Principal component analysis (PCA) of the m^6^A fraction in those conserved m^6^A sites across all tissues in rice and *Arabidopsis*. The modification levels of these m^6^A sites were normalized based on its tissue rankings within the respective species. **e** Gene ontology (GO) analysis of genes containing conserved m^6^A sites. One-sided Fisher’s exact test. Adjusted *P* values using the linear step-up method. **f,** **g** The ratios of standard deviation to mean for m^6^A levels across different tissues in rice (**f**) and *Arabidopsis* (**g)** are presented for various transcript regions: 5′ UTR, CDS and 3′ UTR. Generally, both species exhibit reduced variance from the 5′ UTR to 3′ UTR. Notably, rice showed a gradually reduced variance across the gene structure, while *Arabidopsis* showed rather stable variance in the CDS region followed by a sharp reduction in the 3′ UTR. Source data are provided as a Source Data file.

Next, we investigated the evolutionary conservation of mRNA m^6^A modification in *Arabidopsis* and rice orthologous gene pairs. We found a total of 12,359 pairs of conserved m^6^A sites (Supplementary Data 2) in orthologous genes, while 108,856 and 226,673 m^6^A sites were only identified in *Arabidopsis* and rice, respectively (Fig. 3a). Interestingly, the average m^6^A fractions of unique m^6^A sites are much higher than the rice-*Arabidopsis* conserved sites in rice **(**Supplementary Fig. 4a) and *Arabidopsis* **(**Supplementary Fig. 4b). Amongst the conserved m^6^A site pairs, 7,734 pairs of conserved m^6^A sites exist within the same motif sequence (Supplementary Data 3, Supplementary Fig. 4c and Fig. 3b). The fractions of the conserved m^6^A sites within homologous genes are weakly correlated (Fig. 3c) and vary among different tissues (Fig. 3d and Supplementary Fig. 4d), suggesting that the modification levels of conserved m^6^A sites are tissue dependent even though they are universal among all the tissues. Since the presence of m^6^A is critical for normal plant development^19–21,52^, we explored functional insights about the rice-*Arabidopsis* conserved m^6^A sites. GO analysis showed that genes with conserved m^6^A sites are significantly enriched in stimulus response and plant development-related pathways, such as the chloroplast, photosynthesis, photomorphogenesis, embryo development, shoot morphogenesis, flower development, leaf development, root development, and ovule development (Fig. 3e). For example, the light harvest related genes, *CAB3*, *Lhca5*, *LHCA3*, and *LHB1B2*; root epidermal cell differentiation and root hair cell differentiation-related genes, *POM1*, *GN*, *UBC36*, *GCS1*, *UBC35*, *GEM*, SCN1, and *MRH1*; flower development genes *PFT1*, *PS1*, *RDR6*, *DCL4*, *ARF8*, and *MET1* are all conserved in their m^6^A methylation sites between rice and *Arabidopsis*. Overall, these results provide a foundation for future studies to explore the potential roles of m^6^A under evolutionary pressure in plants.

Despite the evolutionarily conserved m^6^A modification sites across rice and *Arabidopsis*, the metagene profile showed some differences, especially regarding the distribution of the m^6^A sites in the 3′UTR region. To illustrate these differences, we combined all the m^6^A sites within different tissues in rice and *Arabidopsis*, respectively. We calculated the ratio of standard deviation to the mean of m^6^A site counts. We observed that in general, the reduced variance from 5′ UTR to 3′ UTR in rice (Fig. 3f) and *Arabidopsis* (Fig. 3g), while the variance in the 5′ UTR region is fluctuant (Fig. 3f, g). Despite the similarity, rice showed gradually reduced variance across the gene structure, while *Arabidopsis* showed rather stable variance in the CDS region followed by a sharp reduction in variance in the 3′ UTR (Fig. 3f, g). Together these results may suggest differential m^6^A deposition regulations, especially in the CDS and 3′ UTR regions between rice and *Arabidopsis*.

### Divergent paradigms governing m^6^A deposition in plant genomes

Earlier research has elucidated the distribution patterns of m^6^A in both mammalian and plant genomes^38,49,53,54^, suggesting that m^6^A is predominantly enriched in the last exon and long internal exons^53,54^. This distribution is additionally shaped by the underlying exon architecture and is regulated by the Exon Junction Complex (EJC)^55–57^. We categorize these unique distribution patterns into three basic rules: the “long exon,” “last exon,” and “exon structure” rules. Using these quantitative m^6^A sites at single-base resolution, we investigated whether these rules are conserved across the plant kingdom. It’s worth noting that exons in the human genome are generally longer than those in rice and *Arabidopsis* (Supplementary Fig. 5a-c), which could lead to differences in m^6^A distribution both per exon and per sliding window. To improve the accuracy of our measurements, we introduced two metrics: “m^6^A density,” which normalizes the total m^6^A level within each exon by its length, and “m^6^A likelihood,” which normalizes the m^6^A level within each sliding window by the pileup coverage of the exons in that window (Supplementary Fig. 5d).

**Fig. 4:**
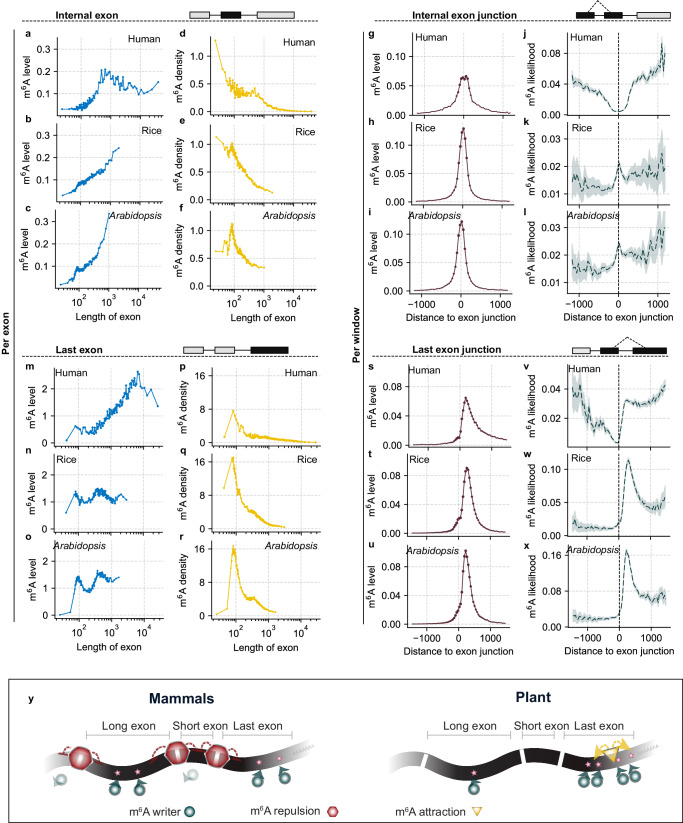
Divergent rules governing m^6^A deposition in plant and mammalian genomes. **a-c** Internal exons of human (**a**), rice (**b**), and *Arabidopsis* (**c**) transcripts were grouped into 100 bins of equal size based on their length, and the average m^6^A level for each bin was plotted against exon length, represented by blue dots. **d**-**f** ‘m^6^A density’ of each bin of the internal exons of human (**d**), rice (**e**), and *Arabidopsis* (**f**) transcripts were shown against exon length, represented by yellow dots. m^6^A density was calculated as the m^6^A level within each exon, normalized by its length and multiplied by 1,000. **g**-**i** All internal exons were aligned at their internal exon junction sites in human (**g**), rice (**h**), and *Arabidopsis* (**i**) genomes, and the overall m^6^A level per sliding window in the flanking regions was shown against the distance to exon junction sites, represented by brown dots. **j**-**l** Distribution of ‘m^6^A likelihood’ near the internal exon junction in human (**j**), rice (**k**), and *Arabidopsis* (**l**) transcripts were shown in dark green line, with 95% confidential intervals shadowed. **m**-**o** Similar to panel (**a**-**c)** but the average m^6^A level per exon in the last exons of human (**m**), rice (**n**), and Arabidopsis (**o**) genomes were shown. **p**-**r** Similar to panel (**d**-**f)** ‘m^6^A density’ in the last exons of human (**p**), rice (**q**), and *Arabidopsis* (**r**) genomes. **s**-**u** Similar to panel (**g-i)** m^6^A level per sliding window flanking last exon junction site in human (**s**), rice (**t**), and *Arabidopsis* (**u**) genomes. **v**-**x** Similar to panel (**j**-**l)** Distribution of ‘m^6^A likelihood’ near the last exon junction in human (**v**), rice (**w**), and *Arabidopsis* (**x**) genomes. **y**, Diagram showing the inhibition mode in humans and activation mode in plants contributes to distinct m^6^A distribution pattern. For **j-l** and **v-x** data are presented as median values.

We re-analyzed single-base mRNA m^6^A data from mammalian samples, specifically from the HeLa cell line^36^, and compared them with data from rice and *Arabidopsis*. We found that the m^6^A level per exon generally increases with internal exon length when that length is under 1000 nucleotides (nt) in both human and plant genomes (Fig. 4a-c and Supplementary Fig. 5e-g). This pattern does not hold for internal exons longer than 1000 nt in humans. Rice and *Arabidopsis* do not display this trend, largely because they have extremely rare internal exons exceeding 1000 nt (Fig. 4a-c). Examining “m^6^A density,” we observed an inverse correlation with internal exon length in both humans and plants (Fig. 4d-f and Supplementary Fig 5h-j). This suggests that m^6^A modifications accumulate more slowly than the exon length increases. Interestingly, overall m^6^A density is higher in plants than in humans, with a peak at around 100 nt in exon length. This indicates that excessively long or short exons could reduce m^6^A modification more effectively in plants.

To estimate the probability of m^6^A modifications, we aligned all exons at their junction sites and calculated the m^6^A levels per sliding window for both humans and plants. Regions closer to these junction sites typically have higher coverage of exons, resulting in increased m^6^A levels (Fig. 4g-i). However, when normalized by the coverage (Supplementary Fig. 5q-s), “m^6^A likelihood” showed suppression behaviors at these junction sites in the HeLa cell line, consistent with prior mammalian studies. In contrast, this pattern was not observed in rice and *Arabidopsis*; instead, we found a slight inverse trend. This divergence suggests that the EJC complex may passively suppress m^6^A deposition in mammalian cells but not in plants. The opposite trend raises the intriguing question of whether the “exon structure” rule governing m^6^A distribution is universally conserved in plants. Active m^6^A deposition pathways may also shape mRNA m^6^A distribution in certain plants.

Regarding the last exons, a clear correlation exists between increasing m^6^A levels per exon and exon length in the HeLa cell line, even when the exon length exceeds 1000 nt (Fig. 4m and Supplementary Fig. 5k). This correlation is notably absent in rice and *Arabidopsis* (Fig. 4n, o and Supplementary Fig. 5l, m). Furthermore, an inverse correlation between ‘m^6^A density’ and exon length was observed for the last exon in both humans and plants (Fig. 4p-r and Supplementary Fig. 5n-p). In contrast to internal exons, m^6^A is more condensed in exons around 100 nt in both humans and plants. Remarkably, the ‘m^6^A density’ in the last exons of rice and *Arabidopsis* is higher than in humans, even though the overall m^6^A level is lower. These findings suggest that while the general pattern of m^6^A enrichment in the last exon is evolutionarily conserved, significant differences exist in how this modification correlates with last exon length across species.

In terms of ‘m^6^A likelihood’ near the last exon junction sites, we observed an asymmetric peak downstream from these sites in both mammals and plants (Fig. 4s-u and Supplementary Fig 5t-v). Similar to internal exons, ‘m^6^A likelihood’ decreases as it approaches the last exon junctions in mammals (Fig. 4v). Notably, specific breakpoints in this distribution pattern occur right at the exon junctions (Fig. 4j, v). This suggests that in mammals, the EJC complex plays a significant role in suppressing m^6^A deposition and that overall m^6^A distribution is regulated by passive processes^40^. In contrast, rice and *Arabidopsis* display a pronounced peak about 300 nt downstream from last exon junction sites, followed by a decrease (Fig. 4w, x). This may indicate an active process driving m^6^A deposition in plants, suggesting enhanced recruitment of m^6^A methyltransferases to the peak region. Given that most stop codons are situated within the last exon, we aligned the exons based on their distance to the stop codon for a more nuanced analysis. We observed a pronounced breakpoint immediately adjacent to the stop codons of rice and *Arabidopsis*, and found that the peak of m^6^A likelihood is closer to the stop codon than to the starting point of the last exon (Supplementary Fig. 5w-y). These findings suggest that the active deposition of m^6^A in plants may be influenced by specific genomic features near the stop codon (Fig. 4y). Collectively, these insights point to potentially significant differences in m^6^A modification mechanisms between plant and mammalian kingdoms, indicating that there may be novel mechanisms for plant mRNA m^6^A deposition yet to be explored.

### m^6^A modification enhances mRNA stability and translation mainly through 3′ UTR in *Arabidopsi*s seedling

**Fig. 5:**
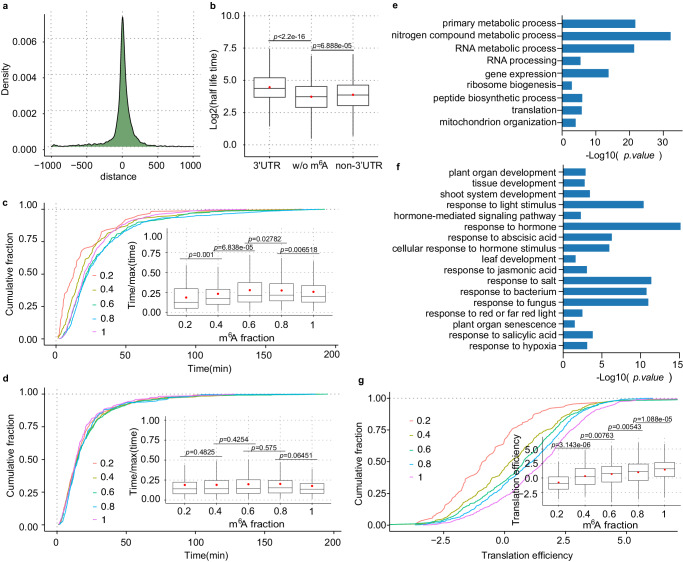
Impact of m^6^A modifications on mRNA stability and translation. **a** Density plot displaying the distance between the peak centers of *ECT2* targets identified by CLIP-seq and m^6^A sites identified in *Arabidopsis* seedlings by m^6^A-SAC-seq. **b** Lifetime difference of transcripts with 3′ UTR-only m^6^A modification and non-3′ UTR m^6^A modification compared to transcripts without (w/o) m^6^A modifications. 3′ UTR, *n* = 1,813; non-3′ UTR, *n* = 2,645; w/o m^6^A, *n* = 3,319. **c** Cumulative curves and box plots showing the mRNA lifetime distribution for transcripts with 3′ UTR-only m^6^A modification. Transcripts were grouped into five categories (0,0.2); (0.2, 0.4); (0.4, 0.6); (0.6, 0.8); and (0.8, 1)) based on the sum of their m^6^A fractions. (0,0.2), *n* = 62; (0.2, 0.4), *n* = 228; (0.4, 0.6), *n* = 353; (0.6, 0.8), *n* = 483; (0.8, 1), *n* = 612. **d** Cumulative curves and box plots showing the mRNA lifetime distribution for transcripts with m^6^A modification outside the 3′ UTR. Transcripts were grouped into five categories (0,0.2); (0.2, 0.4); (0.4, 0.6); (0.6, 0.8); and (0.8, 1)) based on the sum of their m^6^A fractions. (0,0.2), *n* = 466; (0.2, 0.4), *n* = 981; (0.4, 0.6), *n* = 566; (0.6, 0.8), *n* = 280; (0.8, 1), *n* = 294. **e** GO enrichment analysis for genes associated with 3′ UTR-only m^6^A sites. **f** GO enrichment analysis of non-3′ UTR m^6^A-associated mRNAs. For **e**, **f** one-sided Fisher’s exact test. Adjusted *P* values using the linear step-up method. **g** Transcripts with 3′ UTR-only m^6^A sites exhibit strong positive correlations with translation efficiency. Transcripts were grouped into five categories (0,0.2); (0.2, 0.4); (0.4, 0.6); (0.6, 0.8); and (0.8, 1)) based on the sum of their m^6^A fractions. (0,0.2), *n* = 88; (0.2, 0.4), *n* = 293; (0.4, 0.6), *n* = 489; (0.6, 0.8), *n* = 620; (0.8, 1), *n* = 749. For **c**, **d** and **g**, the *Arabidopsi*s seedling lifetime data set GSE86361 was used for mRNA decay analysis and *Arabidopsi*s seedling translation efficiency data set GSE206292 was used for translation efficiency analysis. For **b**-**d** and **g** the *p-*value was determined by a one-tailed Wilcoxon rank-sum test. In box plots, the center line represents the median, and the red dot represents the mean. Upper and lower quartiles were the box limits. Source data are provided as a Source Data file.

m^6^A modification promotes mRNA turnover^37^, mainly through 3′ UTR sites in mammals^58–60^. In contrast to mammals, the effects of m^6^A are less clear in plants, while several reports demonstrated that m^6^A modification stabilizes modified mRNAs in *Arabidopsis*^21,27,61–64^. It seems that the role of m^6^A modification in plants differs from that of mammals. To obtain a more accurate correlation between m^6^A level and mRNA turnover transcriptome-wide, we retrieved the public RNA lifetime data in *Arabidopsis* seedlings^65^ and found that m^6^A-modified transcripts tend to have a longer lifetime than the unmodified transcripts^63^ (Supplementary Fig. 6a). Plants have redundant ECT proteins that bind preferentially to m^6^A-modified mRNAs. We next studied the effects on mRNA degradation regulated by the m^6^A reader of ECT2 with the publicly available ECT2 CLIP-seq data^61^ and mRNA lifetime data^65^ of *Arabidopsis* seedlings. We observed considerable overlap between the m^6^A sites and ECT2 targets (Fig. 5a), confirming m^6^A binding by ECT2. ECT2 target genes containing m^6^A sites display higher lifetime than those not bound by ECT2 (Supplementary Fig. 6b), indicating that m^6^A stabilizes mRNA and that m^6^A readers could enhance mRNA stability in *Arabidopsis* seedlings.

We then asked whether the position of m^6^A modification could underlie mRNA stability differences as that in mammals^59,60^. To answer this, we first clustered the m^6^A sites into 3′ UTR-only m^6^A and non- 3′ UTR m^6^A, and found that 3′ UTR-only m^6^A significantly stabilizes mRNA, and only a slight increased lifetime was observed with genes carrying non- 3′ UTR m^6^A compared to genes without (w/o) m^6^A modification (Fig. 5b). This likely suggests a more predominant role of 3′ UTR-only m^6^A in regulating mRNA stability, especially stabilizing the modified transcripts. To further explore the correlation between m^6^A fraction levels and mRNA stability, we divided mRNA carrying the 3′ UTR-only and non- 3′ UTR m^6^A sites into five groups based on m^6^A levels, and found that higher m^6^A fractions are associated with higher mRNA stability in those genes bearing the 3′ UTR-only m^6^A sites (Fig. 5c**)**, while no significant correlations were observed in genes containing non-3′ UTR m^6^A sites (Fig. 5d and Supplementary Fig. 6c). The above results were further confirmed with the mRNA metabolic data from Sorenson, R. S et al. ^66^ (Supplementary Fig. 6d, e). Although different reader proteins can recognize m^6^A in different regions to exert either stabilization or decay function, given that a majority of mRNA m^6^A modification enriches in the 3′ UTR (Fig. 2a, b), this observation may suggest an overall mRNA stabilization effect by m^6^A in plants.

The distinct effects of m^6^A position in controlling mRNA stability prompted us to investigate whether m^6^A position underlies biological function differences. The GO enrichment analysis showed that genes associated with the 3′ UTR-only m^6^A are significantly enriched in general biological pathways like gene expression, RNA processing, and ribosome biogenesis (Fig. 5e), while non-3′ UTR m^6^A-associated mRNAs were enriched in more specific pathways, such as response to hormone, response to fungus, response to salt stress, leaf development, and plant organ senescence (Fig. 5f). In addition, we also observed a positive correlation between m^6^A levels and translation efficiency in transcripts modified with 3′ UTR-only m^6^A (Fig. 5g), but no significant correlations were noticed in transcripts bearing non-3′ UTR m^6^A sites (Supplementary Fig. 6f). These observations suggest more diverse effects of m^6^A in plant, affected by its location and downstream binding proteins.

### m^6^A installed by MTA in the chloroplast transcriptome in *Arabidopsis* seedlings

**Fig. 6:**
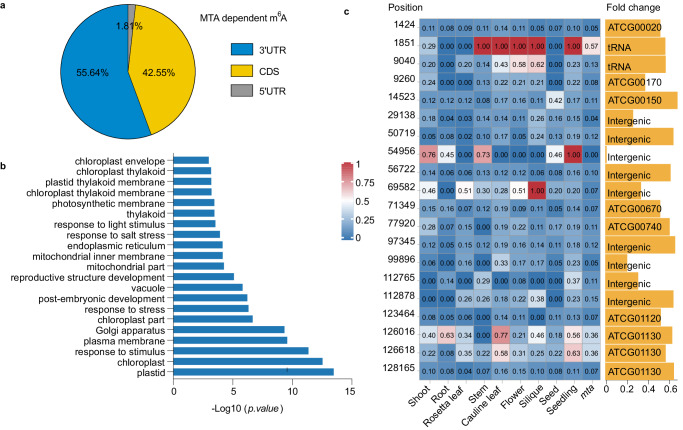
The MTA-mediated m^6^A modification in chloroplast transcriptome of *Arabidopsis* seedlings. **a**, Distribution of MTA-dependent m^6^A sites along transcripts in *Arabidopsis* seedlings. **b**, GO enrichment analysis of mRNAs containing MTA-dependent m^6^A sites. One-sided Fisher’s exact test. Adjusted *P* values using the linear step-up method. **c**, Methylation level heatmap of the chloroplast-encoded genes across different tissues. The position of m^6^A site on chloroplast-encoded genes was shown. m^6^A levels and fold change were also shown. Source data are provided as a Source Data file.

We next profiled the m^6^A sites in *Arabidopsis mta* mutant seedlings (Supplementary Fig. 6g, h) using m^6^A-SAC-seq and compared the m^6^A sites with those of WT (col) seedlings. A total of 14,125 MTA-dependent m^6^A sites within 2,894 RNAs were identified in WT. The methylation levels of these m^6^A sites were noticeably reduced (10,505 m^6^A sites) or completely abolished (3,621 m^6^A sites) in *mta* compared with WT; these m^6^A sites were hereafter defined as MTA-dependent m^6^A sites. We noticed that the MTA-dependent m^6^A sites tend to be more preferentially located in the 3’ UTR region than CDS and 5’ UTR regions (Fig. 6a). GO enrichment analysis showed that mRNAs containing these m^6^A sites are mostly enriched in stimulus response, chloroplast, photosynthesis membrane and post-embryonic development (Fig. 6b). This is consistent with previous studies in *Arabidopsis* that MTA is involved in response to salt stress^26^, blue light response^21^ and embryonic development^15^. Notably, m^6^A levels of chloroplast (538 genes) and photosynthesis membrane (59 genes) related transcripts were significantly reduced in *mta*, among which twenty m^6^A sites showed dramatically reduced m^6^A levels in the chloroplast transcriptome (Fig. 6c). The methylation levels of the chloroplast encoded transcripts vary in different tissues (Fig. 6c), suggesting dynamic MTA-dependent m^6^A modifications in the chloroplast transcriptome across *Arabidopsis* life cycle.

Next, we observed overall reduced translation efficiency in the *mta* mutant compared to that of WT using previously published datasets^26^ (Supplementary Fig. 6i). However, similar numbers of genes with upregulated translation efficiency (332 genes, fold change >2, *p* < 0.05) and downregulated translation efficiency (257 genes, fold change <0.5, *p* < 0.05) were observed in *mta* mutant relative to WT control. The effect of m^6^A installed by MTA on translation efficiency appear to be heterogeneous in *Arabidopsis* seedlings, resembling to that observed in mammals^67^. Perhaps consistently, the above differentially translated genes are also enriched in distinct GO terms. Genes with upregulated translation efficiency are mainly enriched in general pathways like the ribosome and nucleolus (Supplementary Fig. 6j), while genes with downregulated translation efficiency are specifically enriched in the chloroplast and photosynthesis membrane (Supplementary Fig. 6k), such as genes of *FIBRILLIN 4* (*FIB4*) and *SMO2*^68^. Collectively, MTA deposits m^6^A modifications both in the nuclear and chloroplast transcriptomes, which regulate photosynthesis.

### Light-induced feedback regulation of the circadian clock through m^6^A

**Fig. 7:**
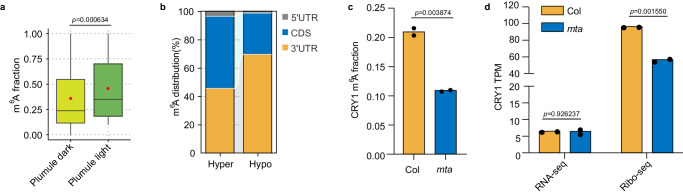
Light responsive regulation through m^6^A in rice and *Arabidopsis.* **a** Light increased the m^6^A fractions in the plumule of rice. Data are means ± SD, *n* = 2. The *p-*value was determined by a one-tailed Wilcoxon rank-sum test. **b** Relative distribution of m^6^A sites across 5′ UTR, CDS and 3′ UTR regions. **c** Reduced m^6^A fraction in *CRY1* was observed in *mta* mutant. Data are means, *n* = 2. **d** Decreased translation efficiency of *CRY1* in the *mta* mutant. Translation efficiency data set GSE206292 was used for the analysis. Data are means, *n* = 2. Student *t*-test was used to determine the statistic difference. Source data are provided as a Source Data file.

m^6^A methylation of mRNAs regulates the circadian clock in both plants^21^ and mammals^69^. To further probe the light effect on rice m^6^A methylation, we germinated rice seeds under dark (24 h of dark per 24 h) and light conditions (16 h of light per 24 h), respectively. Plumules under dark and light conditions at 3 days after germination were studied, respectively, by using m^6^A-SAC-seq (Fig. 7a). Our data revealed a pervasive increase of m^6^A methylation levels (23,253 hypermethylated and 2,607 hypomethylated m^6^A sites) under light compared to dark conditions (Supplementary Fig. 7a-c). The light-induced m^6^A sites are largely outside of 3′ UTR (Fig. 7b). GO enrichment analysis revealed that genes containing hypermethylated m^6^A sites are highly clustered in stimulus response, including light and hormone stimulus (Supplementary Fig. 7d). Noticeably, light significantly increased the m^6^A modification levels of photoreceptor transcripts, for example, *PHYA* (Chr3: 29172686; CDS; AGATA), *PHYB* (Chr3: 11021272; CDS; AGATA), *PHYC*, (Chr3:31007707;CDS; GGACA), *CRY1a* (Chr2: 21976854;5’ UTR; AGAGC), and *CRY2* (Chr2:24921916; 3’ UTR; AAACT). Thus, m^6^A methylation levels of circadian clock genes within their transcripts were notably updated by light in rice, as the case observed in mammals^70^. Moreover, in the aforementioned *mta* mutant of *Arabidopsis*, a decreased m^6^A fraction of *CRY1* in the 3′ UTR (Chr4:5727183; AAACA; 3′ UTR) was observed, potentially resulting in reduced translation efficiency, suggesting that MTA regulates CRY1 translation through m^6^A deposition (Fig. 7c, d). Given that light-induced phase separation of CRYs modulates MTA activity in plants^69^, and MTA controls m^6^A modification on *CRY* transcripts to regulate CRY translation, there appears to be a feedback loop of epitranscriptome-translation regulation of the circadian clock in plants.

## Discussion

m^6^A methylation of mRNA plays critical roles in both plant and mammal development as well as signaling and stimulation responses^15,20,38,71,72^. Previous RNA m^6^A studies in plants lack base-resolution, precision and modification stoichiometry information^5,23,48,63^. Benefit from the development of m^6^A sequencing at single-base resolution in mammalian transcriptome using the m^6^A-SAC-seq method for the first time^36,37^, we report here comprehensive maps of m^6^A at single-base precision with stoichiometry information in eight rice tissues and nine *Arabidopsis* tissues spanning their life cycle. We uncovered high-confidence, single-base resolution m^6^A sites across rice and *Arabidopsis* tissues, providing in-depth resources for future investigations of m^6^A functions in rice and *Arabidopsis*.

Evolutionarily conserved m^6^A modification sites in orthologous gene pairs of *Arabidopsis* and rice were identified. Orthologous genes containing conserved m^6^A sites are significant for tissue development, photosynthesis and stimulus response, and might be selected under evolutionary pressure. Despite these conserved m^6^A sites in orthologous genes, we observed different m^6^A distribution patterns in 3′ UTR between rice and *Arabidopsis*. Furthermore, although a positive correlation between the total level of m^6^A and the internal exon length was observed, different from that in mammals, no such correlation was seen regarding to the last exon in plants. While the mRNA m^6^A distribution in mammals appears to be shaped by repressive pathways mediated through the exon junction complexes or other protein factors^55–57^, plants, by contrast, also rely on active installation to control m^6^A deposition. In particular, our results suggest an active m^6^A deposition process occurring near the stop codon in plant mRNAs. This suppression-activation dual deposition regulation potentially depicts m^6^A distribution patterns across species. We and others have shown that m^6^A suppression is mediated by EJC in mammals^55–57^, the suppression-activation model predicts RNA-binding proteins that may recruit m^6^A writers and direct m^6^A deposition near the stop codon in plant mRNAs. These observations suggest an as-yet-unknown mechanism that regulates m^6^A distribution in plant transcriptomes, which requires future in-depth investigations.

A majority of plant m^6^A modification resides in 3′ UTR from our analyses. Arising evidence in plants suggested that m^6^A modification stabilizes mRNA^21^ although mammalian m^6^A modification tends to destabilize the modified mRNA^37^. Based on our single-base resolution results in *Arabidopsis* seedlings, we observed a positive correlation between m^6^A methylation level and mRNA stability for 3’ UTR m^6^A sites, confirming an overall mRNA stabilization effect by m^6^A in certain plant tissues. Consistently, the m^6^A sites bound by its reader protein ECT2 exhibit significantly elevated half lifetime as compared with transcripts not bound by ECT2, indicating the presence of m^6^A reader protein to enhance mRNA stability in plants^61,62^. In addition, m^6^A methylation installed by MTA can either promote or reduce translation efficiency in a pathway-dependent manner, resembling observations made in mammals^60^. Therefore, m^6^A modification stabilizes modified transcripts, with reader proteins stabilizing the bound mRNA in *Arabidopsis* seedlings. Translation effects can be complex and context-dependent but an overall translation promotion effect in *Arabidopsis* seedling was suggested from our data.

In conclusion, these base-resolution and quantitative m^6^A modification maps across rice and *Arabidopsis’* life cycle have filled a pronounced gap in plant research. The comparative analysis of single-base m^6^A maps between humans and plants reveals a suppression-activation dual regulation model in shaping m^6^A distribution patterns in different species.

## Methods

### Plant material

Col-0 accession of *Arabidopsis thaliana and japonica* rice (*Oryza sativa*) cultivar Nipponbare were used in this study. *Arabidopsi*s, plants were grown at 22 °C with 16 h of light per 24 h. *Arabidopsis* seedlings were harvested after growing on 1/2 Murashige and Skoog medium (MS) plates for 7 d. *Arabidopsis* shoots and roots were harvested after growing on 1/2 Murashige and Skoog medium (MS) plates for 14 d. *Arabidopsis* Rosetta leaves were harvested after growing in soil for 30 d. *Arabidopsis* cauline leaf, flower, stem and silique were harvested after flowering. *Arabidopsis* seeds were collected after the seeds is totally dry. Seeds of *Arabidopsis mta* mutant (*ABI3::MTA/mta*)^20,21^ were sowed in the MS plates, and the *mta* seedlings were harvested at 8-day-old at 22 °C with 16 h of light per 24 h. For rice, plants were grown at 28 °C with 14 h of light per 24 h and 8-day-old, 2-week-old seedlings were harvested. The heading panicles, flag leaf at 10 days after anthesis, endosperms and embryos at 10 days after anthesis were harvested. The plumules under dark and light conditions at 3 days after germination were sampled. The tissue was flash-frozen in liquid nitrogen, ground using a mortar and pestle and stored at −80 °C. Total RNA were extracted using TRIzol™ Reagent (Catalog number: 15596026) according to the manufacturer′s instructions. All the plants were planted in the greenhouse of The Chinese University of Hong Kong.

### mRNA capture from the extracted total RNA

A total of 50 μg total RNA for each of the two biological replicates was used for mRNA capture (Dynabeads mRNA DIRECT Purification Kit (Invitrogen)) following the manufacturer′s instructions with modification. Briefly, 50 ug total RNA was diluted with H_2_O in 100 ul volume and then denatured under 65 °C for 2 min, and immediately put on the ice for exactly 2 min. Next, a total of 100 ul Dynabeads were washed twice with 200 ul lysis/binding buffer provided in the above kit. The washed beads were then eluted in 100 ul lysis/binding buffer, which was further mixed with the denatured total RNA. Then, the sample was put on the rotor to bind for 15 min at room temperature. After binding, wash buffer B was used to wash the beads for twice. 30 ul H_2_O was used to elute the beads and immediately put on the 75 °C for 2 min. The eluted mRNA was collected after magnetic separation. The mRNA capture process was repeated as described above to obtain the more purified mRNA.

### Quantification of m^6^A in RNA by LC–MS/MS

50 ng mRNAs were digested into nucleosides, and the amount of m^6^A was measured by using Agilent 6460 Triple Quad MS–MS with a 1290 UHPLC supplied with a ZORBAX Eclipse XDB-C18 column (UHPLC–QQQ–MS/MS) and calculated based on the standard curve generated by pure standards. For each sample, RNA was digested by using nuclease P1 (NEB) at 37 °C for 2 h. Then, 1 μl of Shrimp Alkaline Phosphatase (rSAP) and 3 μl of 10× rCutsmart buffer (NEB) were added, and the reaction was incubated at 37 °C for 2 h. Samples were then filtered using a 0.22-μm filter (Millipore) and injected into LC–MS/MS. The nucleosides were quantified by using the nucleoside-to-base ion mass transitions of 282 to 150 (m^6^A), and 268 to 136 (A). Quantification was performed in comparison to the standard curve obtained from pure nucleoside standards run on the same batch of samples. The ratio of m^6^A to A was calculated based on the calibrated concentrations.

### m^6^A-SAC-seq library construction

50 ng mRNAs of each replicate were used for the library construction. All these libraries were constructed exactly following the previously published protocols^36,37^. The constructed libraries were sequenced on the Illumina HiSeq sequencing platform in pair-end mode with 150 bp per read.

### m^6^A-SAC-seq data processing

After sequencing, the m^6^A sites were detected using the method of (https://github.com/y9c/m6A-SACseq)^36,37^. The analysis utilized the reference genome downloaded from the Ensemble database, with assembly versions TAIR10 and IRGSP-1.0 employed for Arabidopsis and rice respectively.

### RNA lifetime profiling and translation data analysis

RNA lifetime and translation efficiency data of Arabidopsis Col seedling was downloaded from data set GSE206292 and GSE118462. The translation efficiency data of *mta* mutant in Arabidopsis was also retrieved from GSE206292.

### Conserved m^6^A sites in ortholog genes between rice and *Arabidopsis*

The one-to-one ortholog genes between rice and *Arabidopsis* were first identified. The ortholog genes were then pairwise aligned to obtain the sites on the homolog positions which showed consistent flanking sequence (±1 nt) centered on A sites. The above A sites were defined as the conserved A sites. While the m^6^A modification on conserved A sites for both species are defined as conserved m^6^A sites.

### Gene Ontology (GO) analysis

Functional GO enrichment analysis was performed by web-based toolkit for the agricultural community agriGO v2.038 (http://systemsbiology.cau.edu.cn/agriGOv2/). GO terms with a false discovery rate (FDR) < 0.05 were considered significantly enriched.

### Statistics and reproducibility

All experiments were repeated independently at least twice and showed similar results. GraphPad Prism v.9 and R studio were deployed for the figure plotting.

### Reporting summary

Further information on research design is available in the Nature Portfolio Reporting Summary linked to this article.

### Supplementary information

Supplementary Information

Description of additional supplementary files

Supplementary Data 1

Supplementary Data 2

Supplementary Data 3

Reporting Summary

### Source data

Source Data

## Data Availability

All data supporting the findings of this study are available in the main text or the Supplementary Data. The rice SAC-seq data and *Arabidopsis* SAC-seq data generated in this study have been deposited in the Gene Expression Omnibus database under the GEO numbers of GSE243722 and GSE245738, respectively. Source data are provided with this paper.
