# Supplementary material for: Quantitative profiling of m6A at single base resolution across the life cycle of rice and Arabidopsis
Source: Nat Commun. 2024 Jun 7;15:4881. doi: 10.1038/s41467-024-48941-7 (PMC11161662; doi:10.1038/s41467-024-48941-7)
Supplement: Supplementary file 1 — Supplementary Information [file 41467_2024_48941_MOESM1_ESM.pdf]

Supplementary Figures

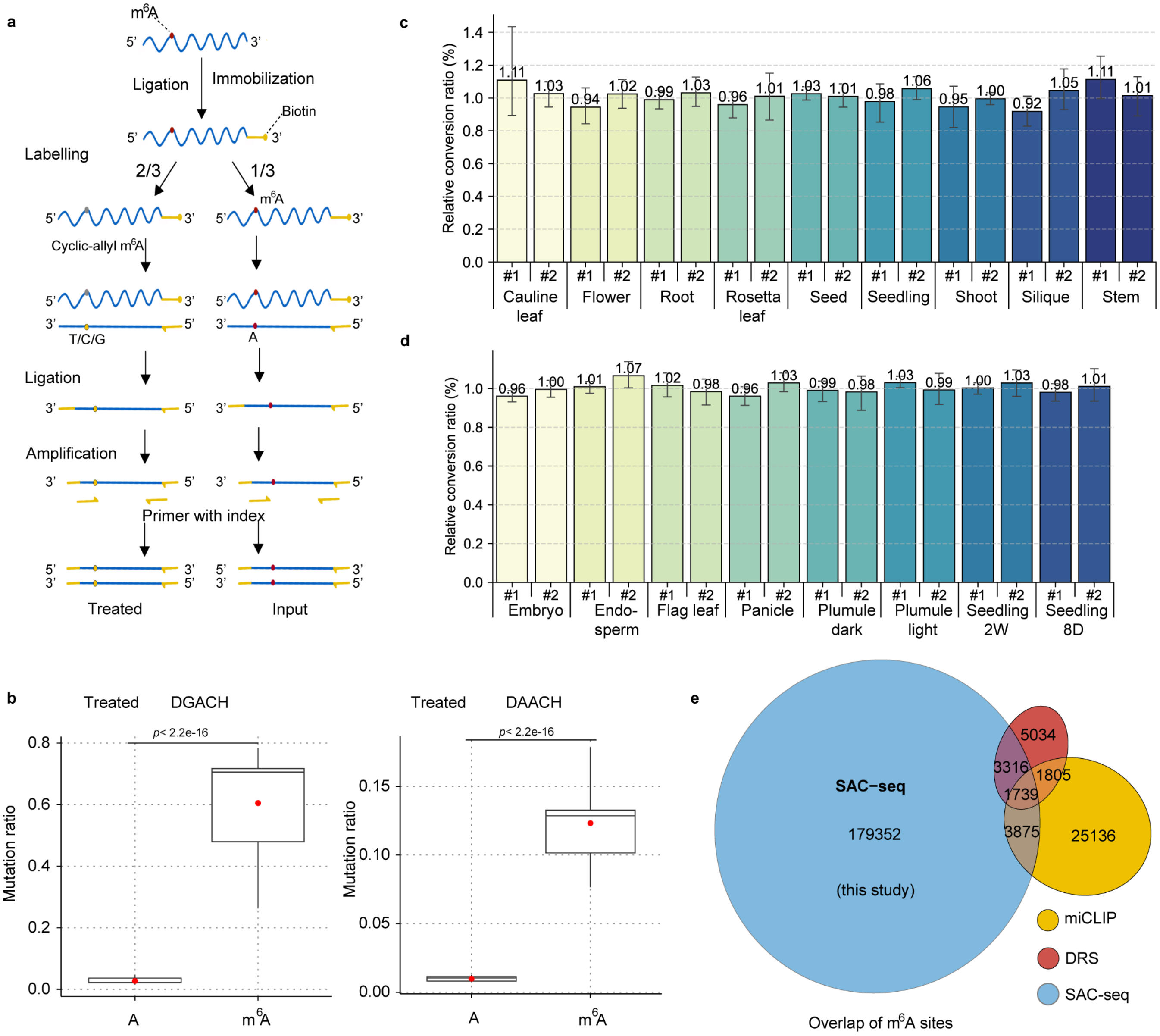

Supplementary Fig. 1: Overview of the quality of m⁶A-SAC-seq data.

a, The schematic diagram illustrating the m^{6}A-SAC-seq protocol. **b**, Mutation ratios of A and m^{6}A sites on spike-in probes with different motifs (n = 256), in treated (MjDim1 labeled) or untreated samples. The median value was marked as the black line in the box plot. GAC motif showed much higher mutation ratio than AAC motif. The *P* value was determined by one-tailed Wilcoxon rank-sum test. **c**,**d**, The relative conversion ratios in Arabidopsis tissues (**c**) and rice tissues (**d**). Two biological replicates were used. Data are means ± SD, *n* = 3. **e**, Venn diagram showing the m^{6}A sites detected by SAC-seq overlapped with those identified through miCLIP and DRS.

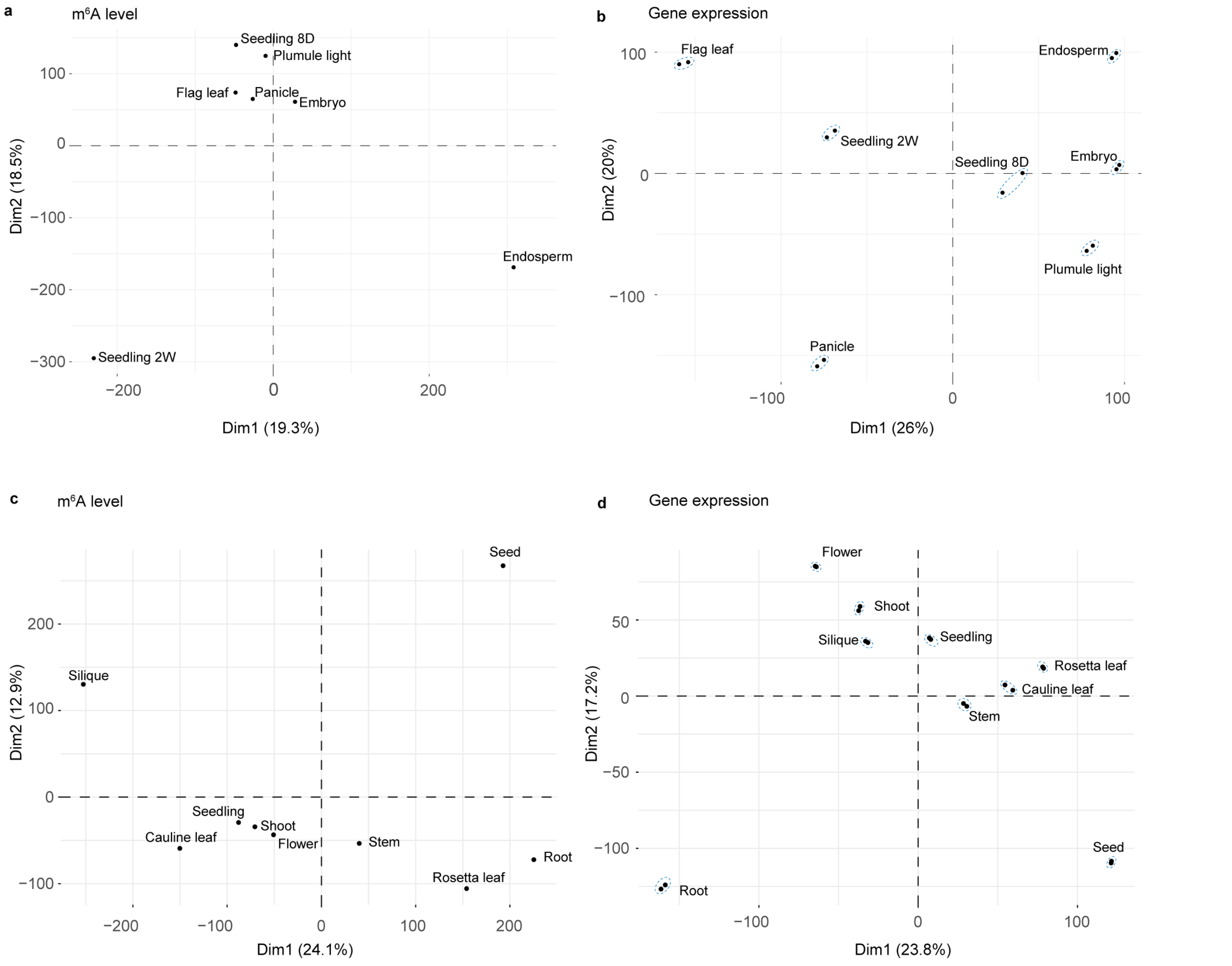

Supplementary Fig. 2: Principal component analysis (PCA) of m^{6}A-SAC-seq data and RNA-seq data. **a,b,** PCA of m^{6}A fractions (**a**) and gene expression (**b**) in rice. **c**, **d**, PCA of m^{6}A fractions (**c**) and gene expression (**d**) in *Arabidopsis*.

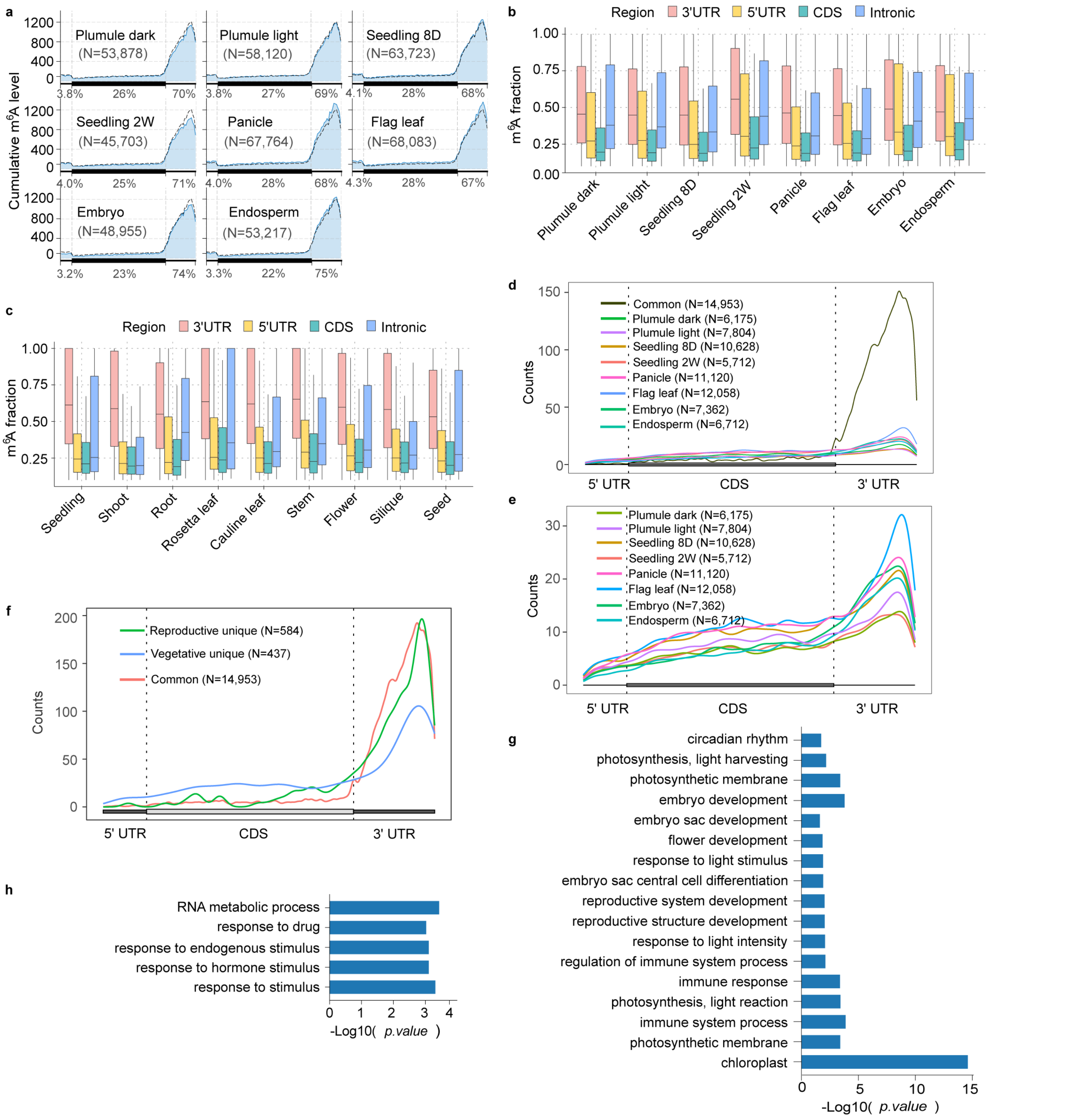

Supplementary Fig. 3: Tissue-specific m^{6}A modification in various tissues of *Arabidopsis*.

a, Metagene profile showing the m^{6}A sites of nine *Arabidopsis* tissues distributed across transcript. The black dash line is the average m^{6}A fraction among the nine tissues. The m^{6}A site number (N) is indicated in the figure. The percentage of m^{6}A fraction distributed in 5′ UTR, CDS, and 3′ UTR regions within different tissues is shown. **b, c,** m^{6}A fraction in different rice tissues (**b**) and *Arabidopsis* tissues (**c**) as showed in the 5′ UTR, CDS, intronic and 3′ UTR regions. **d**, **e**, Metagene profile showing shared m^{6}A sites and tissue unique m^{6}A sites among rice tissues distributed across transcript (**d**), and the metagene profiles of tissue unique m^{6}A sites were also displayed separately in (**e**). The m^{6}A site number (N) is indicated in the figure. **f**, Metagene profile showing common-,

reproductive unique- and vegetative unique- m^6A sites distributed across transcript. Tissues of panicle, embryo and endosperm were combined as the reproductive tissue, while the other tissues were combined as the vegetative tissues**.** Each transcript is divided into three parts: 5′ UTR, CDS and 3′ UTR. The m^6A site number (N) is indicated in the figure. **g**, GO enrichment analysis of genes carrying reproductive unique m^6A modification sites. **h**, GO enrichment analysis of genes containing vegetative unique m^6A modifications. For **g** and **h**, One-sided Fisher's exact test. Adjusted *P* values using the linear step-up method.

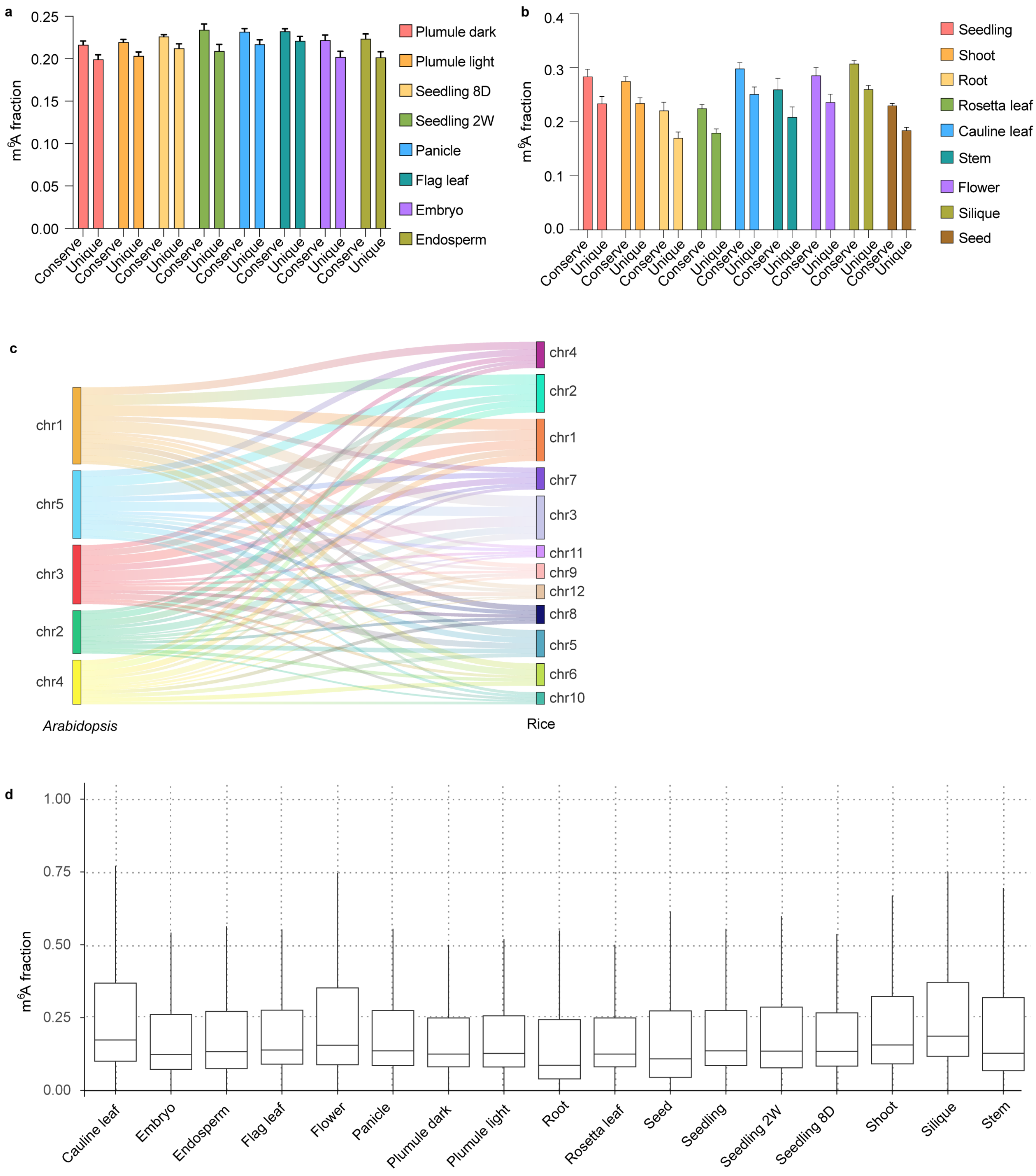

Supplementary Fig. 4: Conserved m^{6}A site pairs across rice and *Arabidopsis* orthologue genes.
a, m^{6}A fractions of rice unique sites compared to the rice-*Arabidopsis* conserved sites in rice. Data are means ± SD, n = 2. **b,** m^{6}A fractions of *Arabidopsis* unique sites compared to the rice-*Arabidopsis* conserved sites in *Arabidopsis*. Data are means ± SD, n = 2. **c**, Sankey plot showing the correlation of these conserved m^{6}A site pairs in orthologue genes of rice and *Arabidopsis*. **d**, m^{6}A fractions of these conserved m^{6}A site pairs in orthologue genes of rice and *Arabidopsis* within different tissues. In box plots, the center line represents the median. Upper and lower quartiles were the box limits.

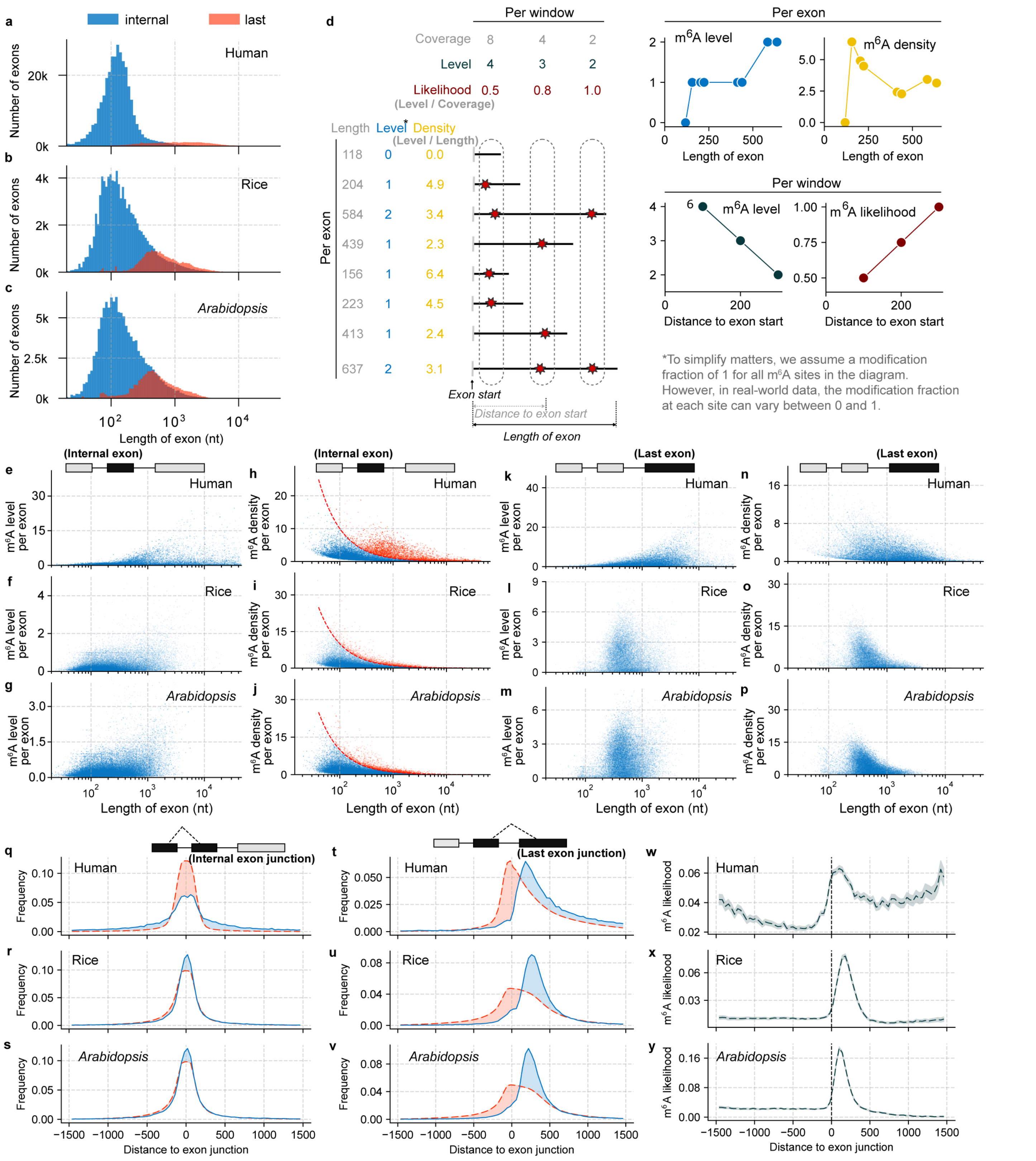

Supplementary Fig. 5: Detailed analysis of m^{6}A levels and exon lengths in plant and mammalian genomes. a-c, Average exon lengths in the genomes of human (**a**), rice (**b**) and *Arabidopsis* (**c**) in regarding to last exons and internal exons. **d**, Diagram outlines the definition of 'm^{6}A density' per exon and 'm^{6}A likelihood' per sliding window. For 'm^{6}A density,' the sum of all detected m^{6}A sites within each exon is normalized by the exon length. The diagram illustrates that the level of m^{6}A per exon may increase with the length of the exon, but the trend in 'm^{6}A density' can decrease if the rate of m^{6}A accumulation is slower than the rate at which exon length increases.

For 'm^{6}A likelihood,' exons are aligned at their junction sites, and the overall 'm^{6}A level' for each sliding window, starting from these junction sites, is calculated. The pileup coverage of these aligned exons is also calculated within each sliding window. 'm^{6}A likelihood' is then defined as the ratio of the 'm^{6}A level' to the exon coverage within the same window. The diagram indicates that 'm^{6}A likelihood' may show an increasing trend even if the 'm^{6}A level' is increasing at a slower rate than the decrease in exon coverage. **e-g**, m^{6}A levels of each internal exon of the human (**e**), rice (**f**) and *Arabidopsis* (**g**) genomes were plotted against exon length. **h-j**, m^{6}A density in the internal exons of human (**k**), rice (**l**), and *Arabidopsis* (**m**) transcripts were shown against exon length, represented by blue dots. m^{6}A density was calculated as the total modification level of all m^{6}A sites within each exon, normalized by exon length and multiplied by 1,000. Long exons with m^{6}A density showing unusually elevated levels of m^{6}A methylation (level per exon > 1) are highlighted in red. **k-m,** Analysis similar to panels **e-g**, but focusing on m^{6}A levels and lengths for last exons. **n-p**, Similar to panel **h-j**, but the m^{6}A density in last exons were shown. **q-s**, Frequency distribution of cumulative exon coverage relative to the internal exon junction sites was plotted in red line, and the frequency distribution of cumulative modification levels for all m^{6}A sites were plotted in blue line. Regions where exon coverage exceeds m^{6}A levels in frequency are shaded in red, and the opposite is shaded in blue. **t-v**, Analysis similar to panels **q-s**, but focusing on the last exons. **w-y**, Distribution of m^{6}A site deposition likelihood near the stop codon in human (**w**), rice (**x**), and *Arabidopsis* (**y**) transcripts. Data are presented as median values.

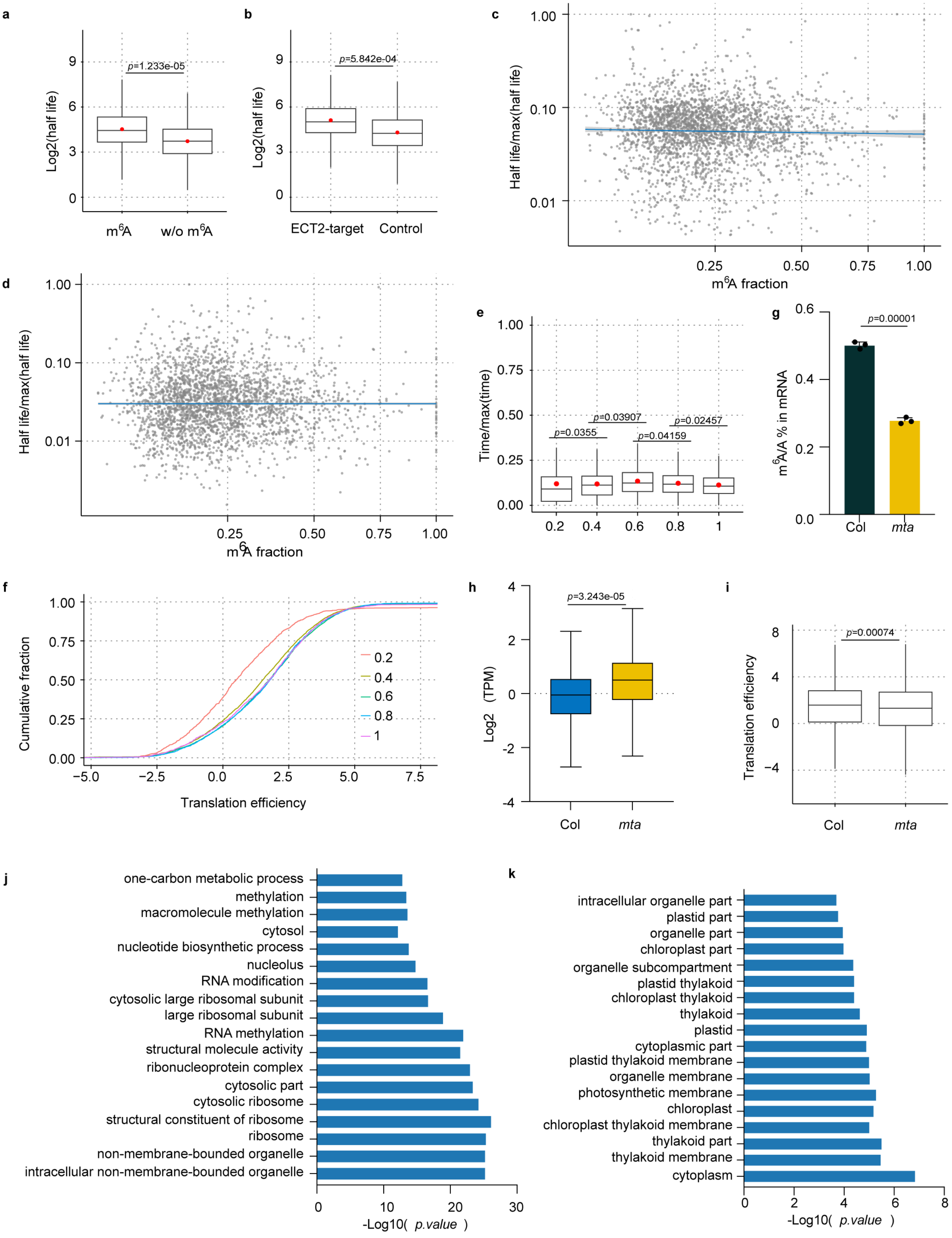

Supplementary Fig. 6: Effects of m^{6}A on the modified RNAs.

a, m^{6}A modified transcripts showed higher lifetime than the unmodified transcripts. m^{6}A, n = 7,77; w/o m^{6}A, n = 3,319; **b**, higher lifetime of ECT2 targets with m^{6}A modification than those not targeted by ECT2. ECT2-

target, n=584; Control, n=13,944. **c,** No significant correlation of non-3′ UTR m^6A modification levels with mRNA lifetime showed in scatter plot. **d**, Scatter plot showing correlation of non-3′ UTR m^6A modifications with mRNA lifetime using mRNA decay data set GSE118462. **e**, Box plots showing the lifetime distribution of 3′ UTR m^6A modification with mRNA lifetime using mRNA decay data set GSE118462. Transcripts were grouped into five categories (0,0.2); (0.2, 0.4); (0.4, 0.6); (0.6, 0.8); and (0.8, 1)) based on the sum of their m^6A fractions. (0,0.2), n=76; (0.2, 0.4), n=265; (0.4, 0.6), n=463; (0.6, 0.8), n=588; (0.8, 1), n=710. **f**, Correlation of transcripts modified by non 3'UTR m^6A sites with translation efficiency. Transcripts were grouped into five categories (0,0.2); (0.2, 0.4); (0.4, 0.6); (0.6, 0.8); and (0.8, 1)) based on the sum of their m^6A fractions. (0,0.2), n=1,003; (0.2, 0.4), n=4,319; (0.4, 0.6), n=4,819; (0.6, 0.8), n=2,415; (0.8, 1), n=1,418. The *Arabidopsi*s seedling translation efficiency data set GSE206292 was used for the analysis. **g**, *Arabidopsis mta* mutant showed significant reduced mRNA m^6A levels. The m^6A-to-A ratio was determined using calibration standards. Data are means ± SD, n =3. Student t-test was used to determine the statistic difference. **h**, Transcript levels of genes associated with reduced m^6A modifications in *mta* mutant compared with the wild type (Col). **i**, Translation efficiency of *mta* mutant was reduced on average. Translation efficiency data set GSE206292 was used for the analysis. **j**, GO enrichment analysis of genes with upregulated translation efficiency mediated by MTA. **k,** GO enrichment analysis of genes with downregulated translation efficiency mediated by MTA. For **j** and **k**, One-sided Fisher's exact test. Adjusted *P* values using the linear step-up method. For **a**, **b**, and **e-i**, the *P* value was determined by one-tailed Wilcoxon rank-sum test. For box plots of **a**, **b**, **e**, **h**, and **i**, the center line represents the median, and the red dot represents the mean. Upper and lower quartiles were the box limits.

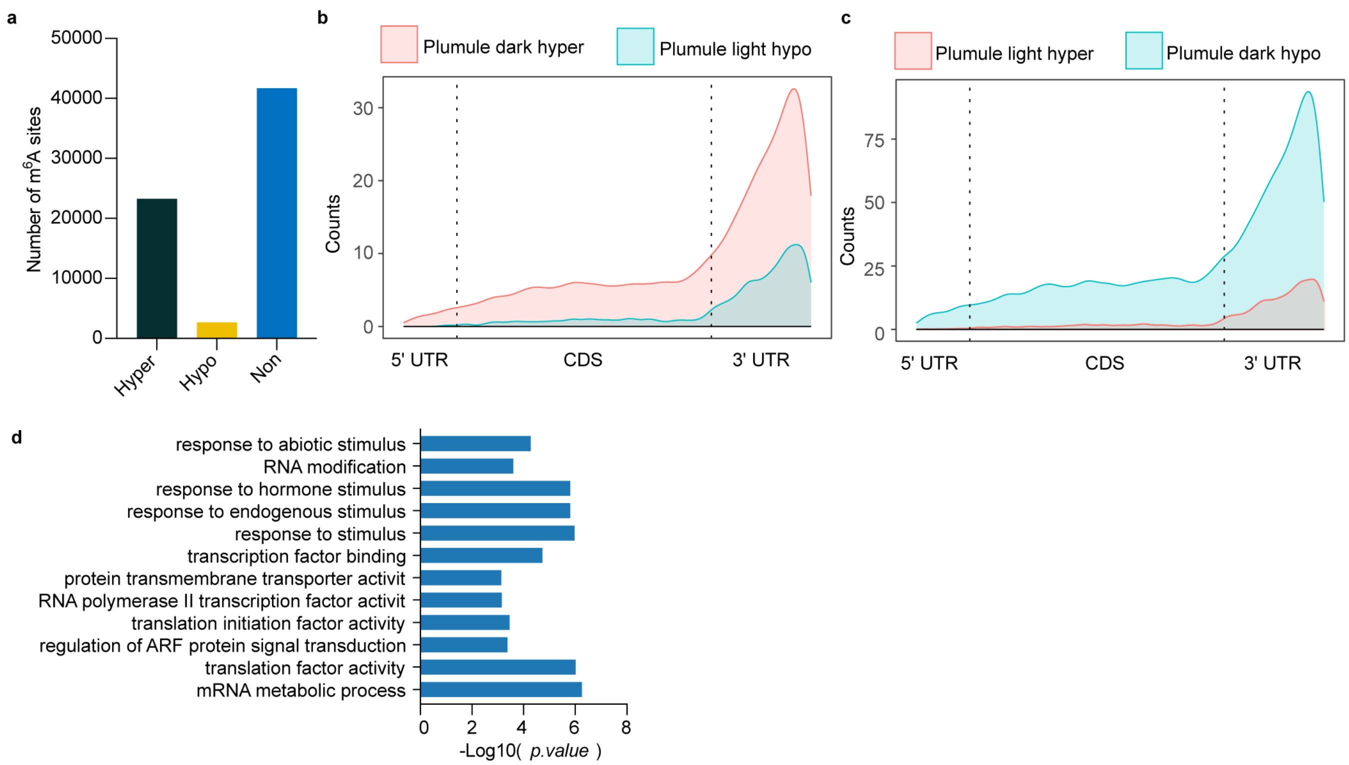

Supplementary Fig. 7: Light responsive m⁶A modification in rice and MTA regulated m⁶A sites in *Arabidopsis*.

a, Number of hypermethylated (hyper) and hypomethylated (hypo) m^6A sites in rice induced by light. **b**,**c**, Metagene profile showing hypermethylated- (**b**) and hypomethylated (**c**) m^6A sites induced by light distributed across transcript. Each transcript is divided into three parts: 5′ UTR, CDS and 3′ UTR. **d**, GO enrichment analysis of genes containing hypermethylated m^6A sites induced by light. One-sided Fisher's exact test. Adjusted *P* values using the linear step-up method.
