# Supplementary material for: Quantitative profiling of m6A at single base resolution across the life cycle of rice and Arabidopsis
Source: Nat Commun. 2024 Jun 7;15:4881. doi: 10.1038/s41467-024-48941-7 (PMC11161662; doi:10.1038/s41467-024-48941-7)
Supplement: Supplementary file 2 — Description of additional supplementary files [file 41467_2024_48941_MOESM2_ESM.pdf]

<u>Description of Additional Supplementary Files</u>

Supplementary Data 1 - Reprocutive unique-, vegetative unique- and conserved m6a sites among rice tissues

Supplementary Data 2 - rice and arabidopsis conserved m6a sites

Supplementary Data 3 - rice and arabidopsis conserved m6a motif
